# A modelling framework based on MDP to coordinate farmers' disease control decisions at a regional scale

**DOI:** 10.1371/journal.pone.0197612

**Published:** 2018-06-13

**Authors:** Anne-France Viet, Stéphane Krebs, Olivier Rat-Aspert, Laurent Jeanpierre, Catherine Belloc, Pauline Ezanno

**Affiliations:** 1 BIOEPAR, INRA, Oniris, Université Bretagne Loire, Nantes, France; 2 CESAER, AgroSup Dijon, INRA, Univ. Bourgogne Franche-Comté, Dijon, France; 3 Normandie Univ, UNICAEN, ENSICAEN, CNRS, GREYC, Caen, France; University of Vermont, UNITED STATES

## Abstract

The effectiveness of infectious disease control depends on the ability of health managers to act in a coordinated way. However, with regards to non-notifiable animal diseases, farmers individually decide whether or not to implement control measures, leading to positive and negative externalities for connected farms and possibly impairing disease control at a regional scale. Our objective was to facilitate the identification of optimal incentive schemes at a collective level, adaptive to the epidemiological situation, and minimizing the economic costs due to a disease and its control. We proposed a modelling framework based on Markov Decision Processes (MDP) to identify effective strategies to control PorcineReproductive andRespiratorySyndrome (PRRS), a worldwide endemicinfectiousdisease thatsignificantly impactspig farmproductivity. Using a stochastic discrete-time compartmental model representing PRRS virus spread and control within a group of pig herds, we defined the associated MDP. Using a decision-tree framework, we translated the optimal policy into a limited number of rules providing actions to be performed per 6-month time-step according to the observed system state. We evaluated the effect of varying costs and transition probabilities on optimal policy and epidemiological results. We finally identifiedan adaptive policy that gave the best net financial benefit. The proposed framework is a tool for decision support as it allows decision-makers to identify the optimal policy and to assess its robustness to variations in the values of parameters representing an impact of incentives on farmers' decisions.

## Introduction

The control of animal diseases is a major concern for the livestock sector. Animal diseases are an important source of vulnerability due to the diversity of their economic impacts [1]. They create substantial shortfalls for farms, by degrading their technical and economic performance (production losses), and lead, for some of them, to the loss of commercial opportunities. The control of animal diseases also implies the allocation of resources, both *ex ante* in terms of surveillance and prevention, and *ex post* to mitigate the sanitary and economic consequences if the disease occurs (*e*.*g*. curative expenditures, disinfection, carcass disposal). These shortfalls and costs induced by animal diseases weigh heavily on the economy of farms and have a wider effect on the competitiveness of animal production chains. Beyond these direct impacts on the livestock sector, animal diseases can have a broader impact on regional and national agricultural economies (animal feed, for example), as well as on firms engaged in the processing of animal products for food.

For infectious diseases, the effectiveness of control measures often depends on the ability to act in a coordinated manner across a group of farms. However, for non-notifiable animal diseases such as Bovine Viral Diarrhoea orPorcine Respiratory and Reproductive Syndrome–most of them being endemic diseases–farmers individually decide whether or not to control the disease, balancing the benefit of implementing or not control measures within their own farm (decentralized decision-making process). As a result, it can lead to a too low proportion of farms under control to ensure disease control at larger (*e*.*g*., regional) scale. Indeed, contagious pathogens spread among farms through numerous transmission pathways such as: animal purchases (*e*.*g*., in paratuberculosis [2]), direct contacts between animals from neighbouring herds (*e*.*g*., in bovine viral diarrhoea [3]), environmental contamination (*e*.*g*., in Q Fever through airborne transmission; [4,5]), equipment shared between farms, animal vectors such as insects (*e*.*g*., in Bluetongue [6]), small mammals, wildlife (*e*.*g*., in tuberculosis; [7]), and movement of persons [8]. Therefore, decision-making at farm level gives rise to externalities that have sanitary and economic consequences to interconnected farms.A farmer who decides to protect his herd against a particular disease by vaccinating or by adopting strict biosecurity measures (*e*.*g*., hygiene, quarantine, etc.) creates a positive externality, in that his action benefits other farmers by lowering the risk of pathogen spread [9]. Conversely, a farmer could behave as a free rider, seeking to benefit from the efforts of his neighbours without bearing the costs [10]. This behaviour generates a negative externality since it contributes to maintaining the disease within a given geographic area. This results in strong interrelationships among individual decisions to control animal diseases at the regional scale. Furthermore, the regional epidemiological situation may vary only if a sufficient number of farmers implements a given control strategy. In a laissez-faire situation, it is likely that the observed outcome of a decentralized decision-making process is not the best outcome for the collective level (pursuit of self-interest does not lead to maximized utility on the aggregate level) [11, 12, 13]. Control decisions should also be understood in a dynamic perspective. Individual decisions regarding disease control are made over time. They vary according to the health statuses of herds to better account for disease spread. Hence, the decision should be adaptive, a key challenge in designing effective schemes. Since the herd health status changes over time, incentives should also vary over time.

Modelling is a powerful tool to assess *ex-ante* adaptive strategies. Often, coordination scenarios are defined in a non-adaptive way (*e*.*g*., [12]). Scenarios are simulated and compared to identify the best ones regarding given criteria. More recently, adaptive approaches have been developed to construct a guideline with rules varying over time and optimizing given criteria. The scope of application encompasses herd management [14, 15], species conservation problems [16, 17], human health management [18, 19, 20], and animal health management [21, 22]. Nevertheless, the issue of adaptive coordination has not been considered yet in animal health management.

Our objective was to facilitate the identification of optimal incentiveschemes at a collective level, adaptiveaccording to theepidemiologicalsituation, and minimizing the economic costs to the community due to a disease and its control. We focused on the collective dimension (i.e., individual decisions are not modelled). We considered a social planner supervising the health management decisions of a group of farmers and proposing collective disease management devices. In the first part of the paper, the modelling framework based on Markov Decision Processes (MDP) is presented.In the second part, this framework is used to identify effective disease control strategies, with application to the control of PorcineReproductive andRespiratorySyndrome (PRRS), an endemicinfectiousdisease thatsignificantly impactsthe productivity of pig farms [23, 24].

## A new modelling framework based on MDP

### Description

We considered that farmers are facing the spread of a non-notifiable endemic animal disease. Farms differ according to the health status of the herd (*e*.*g*., virus-free, infected). For a given herd status, we assumed that all of the farmers are facing similar economic losses due to the disease and similar disease control costs. Farmers’ individual decisions were not explicitly modelled. However, we integrated an epidemiological model describing at each time-step the proportion of herds moving from one health status to another depending on the epidemiological processes that characterize the disease, but also on control measures implemented by farmers.

At the collective level, we considered a social planner [25], whose objective was to improve the welfare of all of the participants in the primary production chain (*i*.*e*., the farmers and the social planner). By taking into account the herd statuses (which derived from epidemiological processes and implemented control measures), the social planner coordinates farmers’ efforts as related to disease control. The social planner’s objective was here to define a sequence of actions over H time-steps {*a*_1_,*a*_2_,…,*a*_*H*_} to improve the economic situation of the primary production chain over this given time horizon (*H* can be finite or infinite). The impact of the actions on the proportion of herds moving from one herd status to another was assumed to be known by the social planner. This proportion is a function of farmers’ compliance with advised measures as well as measure efficacy. These two factors were separated in the framework. Each action had a specific associated cost. The optimization economic criterion used in our model was to seek to minimize, over the given time horizon, the sum of economic costs supported by farmers (*i*.*e*., economic losses due to the disease and disease control costs) and the costs of actions offered by the social planner.

In our framework, the epidemiological model thus dynamically interacts at each time-step with the decision process at the collective level. Herd statuses change over time according to control measures implemented, and thus incentives decided at a collective level.On the other hand, the collective actions are chosen based on farm statuses at each time.

### Model formalization

Markov Decision Processes (MDP) are commonly used to solve sequential decision-making problems under uncertainty [26, 27]. The objective is to provide an optimal policy based on the evolution of the epidemiological situation within a group of herds, and according to the actions taken by the social planner. A glossary of terms is provided in Table 1.

**Table 1:** Glossary of terms used in our modelling framework.

Term	Notation	Meaning
Social Planner		Collective decision-maker who attempts to achieve the best result for all of the parties involved.
State	*s* ∈ *S*	A discrete situation of the system
Action	*a* ∈ *A*	Control variable, here incentive to be used by the social planner
Rewards	*r*(*t*,*s*,*s*′,*a*)	Cost from moving from state *s* at time-step *t* to state *s*′ at time *t* + 1 using action *a* (in k€)
Transitions	*T*(*t*,*s*,*s*′,*a*)	Probability at time-step *t* to move from state *s* to state *s*′ when using action *a*
Objective		The aim targeted by the social planner. It is calculated by summing all of the rewards over the time horizon
Horizon	*H*	Number of successive time-steps (in 6 month periods). At each time-step a decision should be made
Discount factor	*ρ*	Parameter determining how current rewards are valued relative to future rewards
Decision		Determination of the social-planner’s action to take at a given time-step—limited to 1 time-step
Strategy		Succession of decisions over the time horizon
Policy	*π*	Guideline that defines the strategy by indicating at each time-step the action to be taken depending on the state of the system

Formally, our MDP is defined by < *S*, *A*, *T*, *R* > where *S* is the set of states that can be reached by the system, *A*is the set of actions that can be implemented by the social planner, *T*are the probabilities of transitions between states depending on the taken action, and *R*are the rewards associated with the social planner’s actions.

#### States (S)

A state *s* is the distribution of the *N* herds among the *m* possible herd statuses. The set of states*S* is defined by {*s* = (*n*_1_,*n*_2_,…,*n*_*m*_)|*n*_1_ + *n*_2_ + ⋯ + *n*_*m*_ = *N*}.

#### Actions (A)

At each time-step, the social planner chooses one of the available actions *A* = {*a*_1_,*a*_2_,…,*a*_*k*_}, each consisting of a combination of recommendationsand incentives. Each action has a given impact the proportion of farmers implementing advised control measures.

#### Transitions (T)

*T*(*t*,*s*,*s*′,*a*) is the probability to go from state *s* ∈ *S* at time*t*to state *s*′ ∈ *S* at time *t* + 1 when using action *a* ∈ *A*. It depends on the stochastic epidemiological processes and on control measures implemented by farmers.

#### Rewards (R)

The reward for the social planner *r*(*t*,*s*,*s*′,*a*) when usingaction*a*at time *t* is associated with a transition of the system from state *s* at time*t* to state *s*′ at time *t* + 1. At each time-step, rewards (always positive) consist of losses due to the disease and control costs, both depending on the number of herds in each status. Moreover, it may include a cost associated with the action chosen by the social planner, unrelated to herd statuses (*e*.*g*., advising).

### Resolution

Solving a MDP model consists in finding the optimal policy, noted π*. A policy is a function assigning an action for each possible state at each time-step (*π*:*s*_*t*_ → *a*_*t*_). To each policy is associated a cumulative reward. The optimal policy π* is the policy *π* that minimises the expected rewards (costs and losses) cumulated over the time horizon *H*: *E*[∑_*t* = 1,..,*H*_*ρ*^*t*^*r*(*t*,*s*_*t*_,*s*_*t*+1_,*a*_*t*_)|*π*] where *E*[.] is the expectation operator and *ρ*the discount factor (Table 1). The discount factor captures the fact that the social planner overvalues more immediate compared to delayed rewards (the longer a reward is delayed, the more its value decreases). Given this optimal policy, the social planner knows at each time-step which action should be used according to the observed system state. To find π*, the MPD can be solved using the Value Iteration algorithm [26]. Here we used a threshold of 0.1. For a given MDP, this optimal policy always exists [26].

## Case study: Porcine Reproductive and Respiratory Syndrome

PorcineReproductive andRespiratorySyndrome (PRRS) is a major issue for swine industry in most producing countries. For example, annual costs due to PRRS virus have been estimated to be approximately 664 million dollars in the United States in 2011 [24]. Within and between-herd spread of PRRS virus occurs through several transmission routes: purchase of infected pigs, infected semen, introduction from vehicles, people, equipment or supplies, transmission by insects or by air [28]. Depending on the intensity of within-herd virus spread, herds can be classified as negative, positive unstable during the acute phase of infection, or positive stable after stabilization [29]. Measures available to control PRRS in infected herds mostly include biosecurity and immunization through vaccination to limit within-herd spread [30]. To eradicate PRRS virus from the herd, a whole herd depopulation-repopulation is the most effective means but with a high financial cost [30]. Control and eradication programs have been implemented at national or regional levels [31, 32]. Most often they are based on a voluntary adherence byfarmers and require collective organization with coordinated actions of producers and practitioners [30, 32, 33]. Depopulation-repopulation is rarely used in practice due to high associated costs.

### Epidemiological model

**Fig 1:**
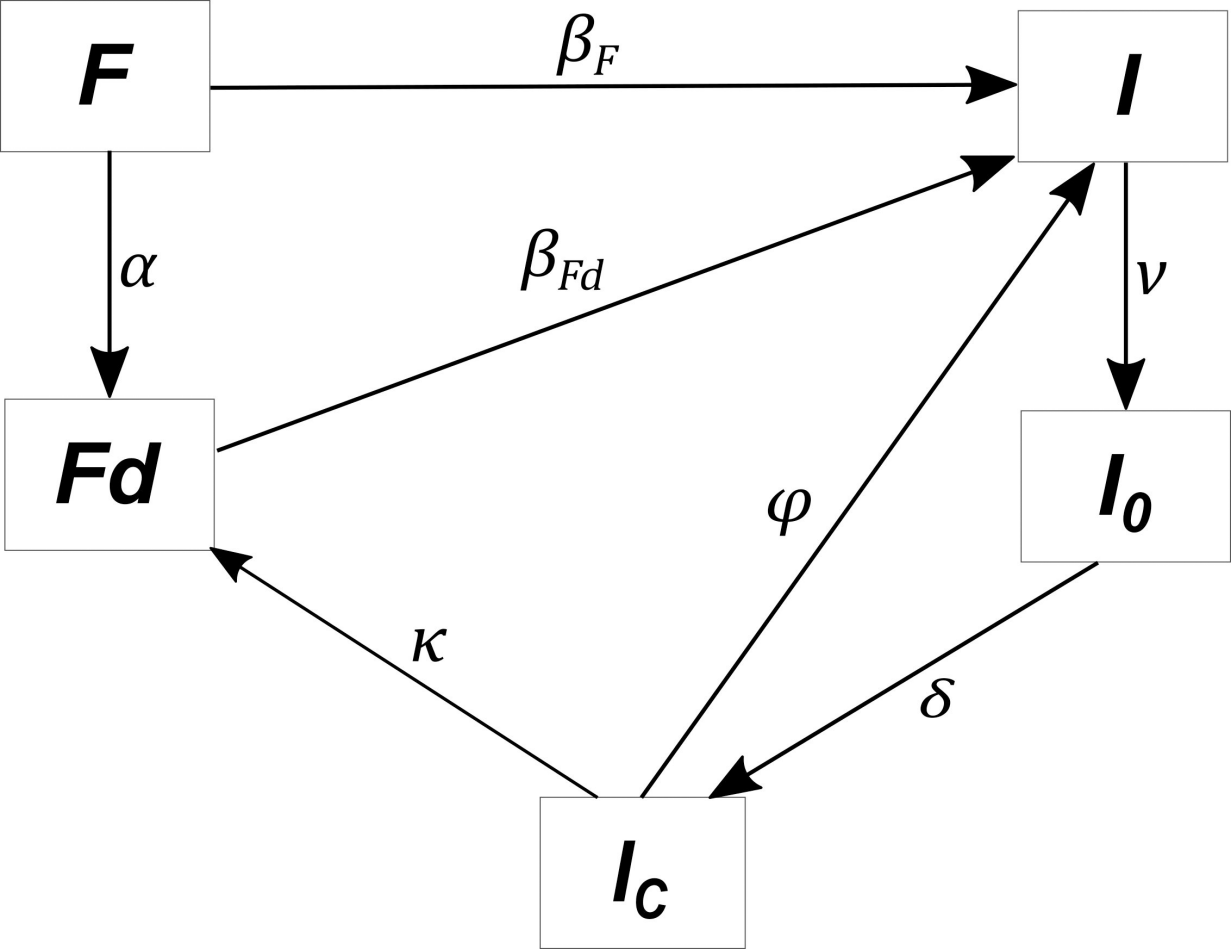
Diagram of transitions between herd statuses. *F* (virus-free herd), *Fd* (virus-free herd with biosecurity measures undertaken), *I* (infected herd without any control), *I*_*0*_ (infected herd with a control measure undertaken but not yet sufficient), *I*_*C*_(controlled infected herd). Letters appearing over the arrows denote transmission probabilities (Table 2, Eqs 1 and 2).

A stochastic discrete-time compartmental model was developed to represent the spread and control of PRRS virus within a group of pig herds. Herds were classified into 5 mutually exclusive statuses combining infection states and individual control measures implemented within the herds: 2 statuses for virus-free herds (*F* and *Fd*) and 3 statuses for infected herds (*I*, *I*_*0*_, and *I*_*C*_; Fig 1). We use throughout the paper similar letters to denote herd statuses and number of herds in these statuses.

**Table 2:** Parameters of the epidemiological model without any action of the social planner: Nominal values and intervals of tested values in the sensitivity analysis of the model.

Symbol	Parameter	Nominal value (per 6 months)	[Min; Max] values
*β*_*I*_	Transmission rate by *I* herds	0.06	[0.045; 0.075]
$\beta_{I_{0}}$	Transmission rate by *I*_*0*_ herds	0.04	[0.03; 0.05]
$\beta_{I_{C}}$	Transmission rate by *I*_*C*_ herds	0.01	[0.0075; 0.0125]
*out*	Transmission rate from outside	0.0001	[0.000075; 0.000125]
*γ*	Protection due to biosecurity in *Fd* herds	0.5	[0.375; 0.625]
*α*	Proportion of *F* herds implementing biosecurity measures	0	[0.075; 0.125]^a^
*ν*	Proportion of *I* herds implementing vaccination and biosecurity	0.1	[0.075; 0.125]
*κ*	Proportion of *I*_*C*_ herds implementing depopulation and biosecurity	0.01	[0.0075; 0.0125]
*φ*	Proportion of *I*_*C*_ herds stopping vaccination and biosecurity	0.05	[0.0375; 0.0625]
*δ*	Proportion of *I*_*0*_ herds with a controlled infection	0.5	[0.375; 0.625]

^a^For sensitivity analysis, we explored no null values

*F* herds are virus-free (*i*.*e*., without any virus circulation). When farmers implement biosecurity measures to prevent virus introduction, virus-free herds *F* become *Fd* (virus-free with biosecurity). Both *F* and *Fd* herds can be infected and then become *I* (with virus spread without control). When control measures to limit virus spread are implemented, infected herds become *I*_*0*_, with a lowered virus spread. When spread is controlled, *I*_*0*_ herds become *I*_*C*_ (controlled infected herds). We assumed that farmers will not stop control measures in *I*_*0*_ herds until reaching status *I*_*C*_. Then, farmers can depopulate, *I*_*C*_ herds then becoming *Fd*. As *I*_*C*_ herds already experienced infection, we assumed that they will maintain biosecurity measures. On the other hand, *I*_*C*_ herds can become *I* again when control measures are stopped without depopulation.

Virus transmissionto *F* and *Fd* herds occurred from herds in *I*, *I*_*0*_, and *I*_*C*_ statuses. Because of the control measures implemented by farmers in *I*_*0*_ and *I*_*C*_ herds, virus transmission is reduced compared to transmission by *I* herds. The virus can also be introduced due to contacts with herds located outside of the modelled group of herds (external risk). Pig production systems are almost closed. However, the external risk is not nil, and, even with a very low value, could impair disease control and thus should be accounted for. Transitions from statuses *F* and *Fd* to status *I* were modelled using a frequency-dependent function [34, 35] (Eq 1). We assumed that the transition rate from *Fd* to *I* is equal to the one from *F* to *I*, weighted by factor *γ*, representing the protection induced by biosecurity (Eq 2).

$$\beta_{F}=\beta_{I}\frac{I}{N}{+\beta }_{I_{0}}\frac{I_{0}}{N}{+\beta }_{I_{C}}\frac{I_{C}}{N}+out$$ (1)

$$\beta_{\mathrm{Fd}}={\gamma \beta }_{F}$$ (2)

where *N* is the total number of herds, and *β*_*I*_, $\beta_{I_{0}},\;\beta_{I_{C}}$, and *out* are the transmission rates to herd *F* from a herd *I*, *I*_*0*_, *I*_*C*_, and from outside, respectively.

The other transitions were defined as constant probabilities representing the proportion of herds in each status that implements the control measures (biosecurity, management practices, vaccination, and depopulation). Parameter values provided in Table 2 correspond to the case where the social planner was assumed not to influence measure implementation in farms. These values were chosen from expert’s opinion assuming a time-step of 6 months.

### MDP model

#### States

The set of states is given by S = {*s* = (*F*,Fd,*I*,*I*_0_,Ic)|*F* + Fd + *I* + *I*_0_ + Ic = *N*}. The number of states is given by the combination (binomial coefficient) $\left(\begin{array}{c} N+4\\ 4 \end{array}\right)$. For a group of 50 herds, there are 316,251 possible states. Only a small proportion of these states is expected to be reachable, reached states depending on initial conditions and parameter values.

#### Actions

We modelled the consequences of the social planner’s action on farmers’ decisions by modifying the value of model parameters related to transitions between herd statuses (Table 3). Four actions were considered: *None*, *Incent1*, *Incent2*, and *Incent3*(Table 3). Transitions between herd statuses (Table 3 in lines) involved one or several measures, or their end, as defined in Table 2. Actions(Table 3 in columns) correspond to a level of incentives to implement a set of measures according to herd status, incentives increasing from *none* to *incent3*. When using action *None*, the social planner did not influence measure implementation on farms but some measures still were assumed to be implemented. When using *incent1*, there was a positive but low incentive to protect virus-free herds and to control infection in infected herds (fewer new infections and returns to *I* state). When using *incent2*, the same measures were advised but with a higher effect, corresponding either to an increasing efficacy of measures or a higher level of farmers’ compliance. For realism, we nevertheless considered that farmers’ compliance was not complete and that for each status, a given proportion of farmers did not implement control measures (we assumed that farmers with infected herds were less reluctant to implement the prescribed control measures than those within virus-free herds). When using *incent3*, measure efficacy and farmers’ compliance was much higher. In addition, depopulation-repopulation of infected herds was considered with no possible return to state *I* from state *I*_*C*_. With such an action, eradication is expected to be achievable.

**Table 3:** Modification of epidemiological parameter with social planner’s actions (in bold) and tested values in the one at a time sensitivity analysis (between brackets [Min, Max]).

Transition	Parameter	Values according to the considered action			
		*None*	*Incent1*	*Incent2*	*Incent3*
*F →Fd*	*α*	**0**	**0.1** [0.075; 0.125]	**0.3** [0.225; 0.375]	**0.75** [0.5625; 0.93725]
*I →I*_*0*_	*ν*	**0.1**	**0.2** [0.15; 0.25]	**0.4** [0.3; 0.5]	**0.7** [0.525; 0.875]
*I*_*C*_*→Fd*	*κ*	**0.01**	**0.01** [0.0075; 0.0125]	**0.01** [0.0075; 0.0125]	**0.1** [0.125; 0.075]
*I*_*C*_*→ I*	*φ*	**0.05**	**0.025** [0.01875; 0.03125]	**0.01** [0.0075; 0,0125]	**0** [0.005]

#### Transitions

Transitions in the decision model were computed based on transitions defined in the epidemiological model (Fig 1). A transition probability given action *a* from state *s*_*t*_ = (*F*_*t*_,*Fd*_*t*_,*I*_*t*_,*Io*_*t*_,*Ic*_*t*_) to state *s*_*t*+1_ = (*F*_*t*+1_,*Fd*_*t*+1_,*I*_*t*+1_,*Io*_*t*+1_,*Ic*_*t*+1_) was defined based on the number of herds moving from one status to another. The probability was not null if, for all statuses, the number of herds at time *t* + 1 is consistent with the one at time*t* given the transitions between herd statuses (Fig 1). For example, as no herd can become *F*, the number of *F* herds at time *t* + 1 should be lower than or equal to the one at *t*. Let us denote:

- *w* the number of herds moving from *F* to *Fd*at time *t*
- *l* the number of herds moving from *F* to *I*at time *t*
- *m* the number of herds moving from *Fd* to *I*at time *t*
- *u* the number of herds moving from *I* to *I*_*0*_ at time *t*
- *y* the number of herds moving from *I*_*0*_ to *I*_*C*_at time *t*
- *x* the number of herds moving from *I*_*C*_ to *Fd*at time *t*
- *z* the number of herds moving from *I*_*C*_ to *I*at time *t*

According to Fig 1, we have

$$\left\{ \begin{array}{c} F_{t+1}=F_{t}-w-l\\ Fd_{t+1}=Fd_{t}+w-m+x\\ I_{t+1}=I_{t}+l+m-u+z\\ Io_{t+1}=Io_{t}+u-y\\ Ic_{t+1}=Ic_{t}+y-x-z \end{array}\right.$$ (3)

Therefore, knowing states *s*_*t*_ and *s*_*t+*1_, we only need to consider potential values of *w*, *x*, and *y*. Indeed, from *w*, we can deduce *l*, from *x* and *w*, we can deduce *m* and from *w*,*x*,and *y*, we can deduce *z* and *u*.

The transition probability when using action*a* was then given by (noting that $\left(\begin{array}{c} n\\ k \end{array}\right)$ is the binomial coefficient):

$$\sum_{x=0}^{min({Ic}_{t},{Fd}_{t+1},{Ic}_{t}-{Ic}_{t+1}+{Io}_{t})}\sum_{w=max(0,{Fd}_{t+1}-{Fd}_{t}+x)}^{min(F_{t}-F_{t+1},{Fd}_{t+1}-x)}\sum_{y=max(0,{Ic}_{t+1}-{Ic}_{t}+x,{Io}_{t}-{Io}_{t+1})}^{min({Io}_{t},{Ic}_{t+1},{Io}_{t}+I_{t}-{Io}_{t+1})}\left[(\begin{array}{c} {Ic}_{t}\\ x \end{array})(\begin{array}{c} {Ic}_{t}-x\\ z \end{array}){\kappa (a)}^{x}\varphi {\left(a\right)}^{z}{\left(1-\kappa (a)-\varphi (a)\right)}^{\left({Ic}_{t}-x-z\right)}\right]\left[(\begin{array}{c} F_{t}\\ w \end{array})(\begin{array}{c} F_{t}-w\\ l \end{array}){\alpha (a)}^{w}{P(F\to I)}^{l}{\left(1-\alpha (a)-P(F\to I)\right)}^{\left(F_{t}-w-l\right)}\right]\left[(\begin{array}{c} {Fd}_{t}\\ m \end{array}){P(Fd\to I)}^{m}{\left(1-P(Fd\to I)\right)}^{\left({Fd}_{t}-m\right)}\right]\left[(\begin{array}{c} {Io}_{t}\\ y \end{array})\delta^{y}{\left(1-\delta \right)}^{\left({Io}_{t}-y\right)}\right]\left[(\begin{array}{c} I_{t}\\ u \end{array}){\nu (a)}^{u}{\left(1-\nu (a)\right)}^{\left(I_{t}-u\right)}\right]$$ (4)

with *P*(*F* → *I*) = 1 − *exp*(−*β*_*F*_) and *P*(*Fd* → *I*) = 1 − *exp*(−*β*_*Fd*_).

In this formula, the first term in square brackets corresponds to the product of two binomials. The first binomial denotes for the *x* herds chosen among *Ic*_*t*_ to go to *Fd*_*t+1*_ with probability $\left(\begin{array}{c} {Ic}_{t}\\ x \end{array}\right){\kappa (a)}^{x}{\left(1-\kappa (a)\right)}^{\left({Ic}_{t}-x\right)}$. The second binomial denotes for the *z* herds chosen among the remaining ones (*Ic*_*t*_ − *x*) to go to *I*_*t+1*_ conditionally they are not going to *Fd*, with probability $\left(\begin{array}{c} {Ic}_{t}-x\\ z \end{array}\right){\left(\frac{\varphi (a)}{1-\kappa (a)}\right)}^{z}{\left(1-\frac{\varphi (a)}{1-\kappa (a)}\right)}^{\left({Ic}_{t}-x-z\right)}$. It has to be noted that the probability for herds not to go to *Fd* while going to *I* is one. Once aggregated you end with the sub-formula in square brackets. Similarly, the second term in square brackets corresponds to the probability for *w* herds to go from *F*_*t*_ to *Fd*_*t+1*_ times the probability for *l* herds among remaining ones to go from *F*_*t*_ to *I*_*t+1*_. The last three terms in square brackets are simpler and corresponds respectively to *m* herds going from *Fd*_*t*_ to *I*_*t+1*_, *y* herds going from *I*_*0t*_ to *Ic*_*t+1*_, and *u* herds going from *I*_*t*_ to *I*_*0t+1*_. Then, we sum over all of the possible values for *x*, *w*, and *y*, accounting for constraints on these sums, each being limited by the number of herds that can change of status (*i*.*e*., not more than available in source status at time *t*, not more than needed at time *t*+1 in receiving status, and not more than missing in the source status at time *t*+1 compared to time *t*).

#### Rewards

For the social planner, the choice of any action *a* ∈ *A* except for action *None* induced a fixed cost *Cdiff(a)*. Moreover, at each time-step, the other control costs (C) and losses (L) due to the disease incurred by the social planner were expressed as functions of the number of herds in each herd status or transiting between statuses. We assumed that the magnitude oflosses in controlled infected herds (*L*_*Ic*_) were lower than those in other infected herds (*L*_*I*_).Herds in statuses *I*_*0*_and *I*_*C*_ had costs due to external biosecurity (*Cbe*) and internal biosecurity with vaccination (*Cbi*). Herds in status *Fd* had costs due to external biosecurity (*Cbe*). Finally, a cost related to depopulation *Cdep*concerned*I*_*C*_herds becoming *Fd*(transition flow between the two statuses). For the social planner in state *s*_*t*_ = (*F*_*t*_,*Fd*_*t*_,*I*_*t*_,*Io*_*t*_,*Ic*_*t*_) at time *t* moving to state *s*_*t*+1_ = (*F*_*t*+1_,*Fd*_*t*+1_,*I*_*t*+1_,*Io*_*t*+1_,*Ic*_*t*+1_) at time *t* + 1 when using action *a*, the reward at time*t* was:/)

$$r(t,s_{t},s_{t+1},a)={\mathrm{Cdiff}(a)+Fd}_{t}.Cbe+I_{t}L_{I}+{Io}_{t}(L_{I}+Cbe+Cbi)+{Ic}_{t}(L_{Ic}+Cbe+Cbi)+x.Cdep$$ (5)

As herds were not individually modelled, we used the expected number computed using the transition probabilities given the initial and final states. The values of losses and costs are given in Table 4.

**Table 4:** Loss (L) and cost (C) values at each time-step for the social planner, as used in the computation of rewards (time-step of 6 months).

Parameter	Definition	Nominal value	Other values tested one-at-a-time [Min; Max]	Comments
*L*_*I*_	Losses for *I* and *I*_*0*_ herds	6,550	[4,912.5; 8,187.5]	Applied to *I* and *I*_*0*_ herds
$L_{I_{C}}$	Losses for *I*_*C*_ herd	4,775	[3,581.25; 5,968.75]	Applied to *I*_*C*_ herds
*Cbe*	External biosecurity cost	250	[187.5; 312.5]	Applied to *Fd*, *I*_*0*_ and *I*_*C*_ herds
*Cbi*	Internal biosecurity and vaccination cost	2,300	[1,725; 2,875]	Applied to *I*_*0*_ and *I*_*C*_ herds
*Cdep*	Depopulation cost	100,000	[75,000; 125,000]	Applied to herds moving from *I*_*C*_ to *Fd*
*Cdiff*(1)	Fixed costs–*Incent1*	10	[7.5; 12.5]	Applied if action *Incent1*is retained
*Cdiff*(2)	Fixed costs–*Incent2*	1,000	[750; 1,250]	Applied if action *Incent2*is retained
*Cdiff*(3)	Fixed costs–*Incent3*	20,000	[15,000; 25,000]	Applied if action *Incent3*is retained

As we considered an endemic disease, we looked for optimality in the long run. For the MDP resolution, we fixed an infinite horizon with a discount factor *ρ*of 0.975 per time-step (6 months).

### Model scenarios and analysis

#### Initial conditions

We considered a group of 50 herds. In order to reflect the endemic situation of the disease, an initial state was chosen corresponding to 40% of the herds in a virus-free status (5 in *F* and 15 in *Fd*), 40% of the herds in status *I*_*C*_ (20 herds), and the remaining 20% in statuses *I*(5 herds) and *I*_*0*_ (5 herds). A program was developed in Java language (S1 File) [36] and the model was simulated (5,000 replications) over 100 time-steps of 6 months (50 years) (S2 File).

#### Outputs

Two outputs were of interest to represent the simulation outcomes: the number of virus-free herds representing the clearance level (*F + Fd*) and the number of controlled infected herds (*I*_*C*_). These outputs, corresponding to non-transient states, were regarded as relevant for an endemic disease.

#### Model behaviour when the social planner always chose action *None*

We first calibrated the epidemiological model so that it produced a realistic equilibrium situation over time if the social planner always chose action *None*. The objective was to represent the endemic situation of the disease in production areas. We then checked the effect of uncertainty in model parameters to identify the parameters that need to be accurately determined. In particular, we verified that the model behaviour was appropriately impacted by parameters influenced by actions. We also conducted a global variance-based sensitivity analysis using the FAST sampling design for varying parameters simultaneously ([37]; 100,000 scenarios), assuming a uniform and continuous distribution between minimal and maximal values (Table 2). For each parameter and each model output, we computed the first order and total sensitivity indices using the *sensitivity* package of the *R* software [38].

#### Computed optimal policy

To assess the advantage of using the optimal policy π*, we compared the epidemiological results and the discounted cumulative rewards $\sum_{t=0}^{100}\rho^{t}r(t,s_{t},s_{t+1},a_{t})$ obtained when using π* versus one action consistently (irrespective of the state).

#### Approximated policy

The optimal policy π* consists of a table of 316,251 lines (each line corresponding to a possible state of the system, the whole table thus describing the action to be performed for each of the possible distributions of the 50 herds among the 5 herd statuses). Such atable cannot be easily used under field conditions. To provide the social planner with a simpler but approximated policy made of alimited number of rules, π* was approximated using a decision-tree into π*,^*approx*^, providing the actions to be performed at each time-step according to the observed state of the system. Only 7.4% of the possible states can be reached from our initial state given our parameter values. The approximation of π* was done only using these states, called hereafter available states. A supervised classification approach was performed using the *C*4.5 algorithm [39, 40] available as an*R* package (*RWeka* Package). This method uses as dataset available states and their associated actions as known in the optimal policy and generates errors corresponding to misclassifications (i.e., for a few states, another action than the one given by π* can be predicted by π*,^*approx*^). We evaluated the quality of π*,^*approx*^ by calculating the percentage of misclassifications.

#### Scenario analysis

As model parameters were roughly estimated, particularly transition probabilities for which no observed data were available, we evaluated the effect on π* and epidemiological results of varying costs and losses (16 scenarios; Table 4), transition probabilities (19 scenarios; Table 3), and the level of protection conferred to virus-free herds by biosecurity implementation (*γ*; 2 scenarios; Table 2) one-at-a-time, resulting in a total of 37 scenarios. We restricted analyses to transitions impacted by the social planner’s actions, *i*.*e*. all except *None*.

As a result ofthe high computing time needed for each scenario (nearly 20 hours despite the use of multi-thread programming), we did not investigate interactions among factors. We compared π*,^*approx*^ among scenarios and evaluated a weighted divergence index to account for differences between retained actions in the policy for the available states only (states which can be reached from the initial state). First, we evaluated the frequency of visits of each state for all of the scenariosincluding the reference one (with all nominal values), noted *wg(s)*for state *s*. The divergence index for scenario *i*was the sum of the visit frequencies over all states for which the action implemented for state *s* in scenario *i* was not similar to the action implemented for the same state *s*in the reference scenario: $\sum_{s\in S}wg(s)1_{\left\{a_{i}(s)\neq a_{ref}(s)\right\}}$ where $1_{\left\{a_{i}(s)\neq a_{ref}(s)\right\}}$ is a Boolean. A weighted divergence index equal to 0means that the approximated policies are similar. However, it does not inform about other states. We compared also the cumulative simulated use of each action by defining *use*_*i*_(*a*) − *use*_*ref*_(*a*) with *use*_*k*_(*a*) the cumulative proportion over time of the use of action *a* for scenario *k =* {*i*,*ref*}. For epidemiological results, we compared the median proportion of infected controlled herds (*I*_*C*_) and of non-infected herds (*F + Fd*) after 50 years in scenario *i* and in the reference. Finally, we compared the median discounted cumulative rewardsobtained after 50 years in scenario *i* and in the reference.

## Results

### When the social planner does not influence measures implemented on farms

**Fig 2:**
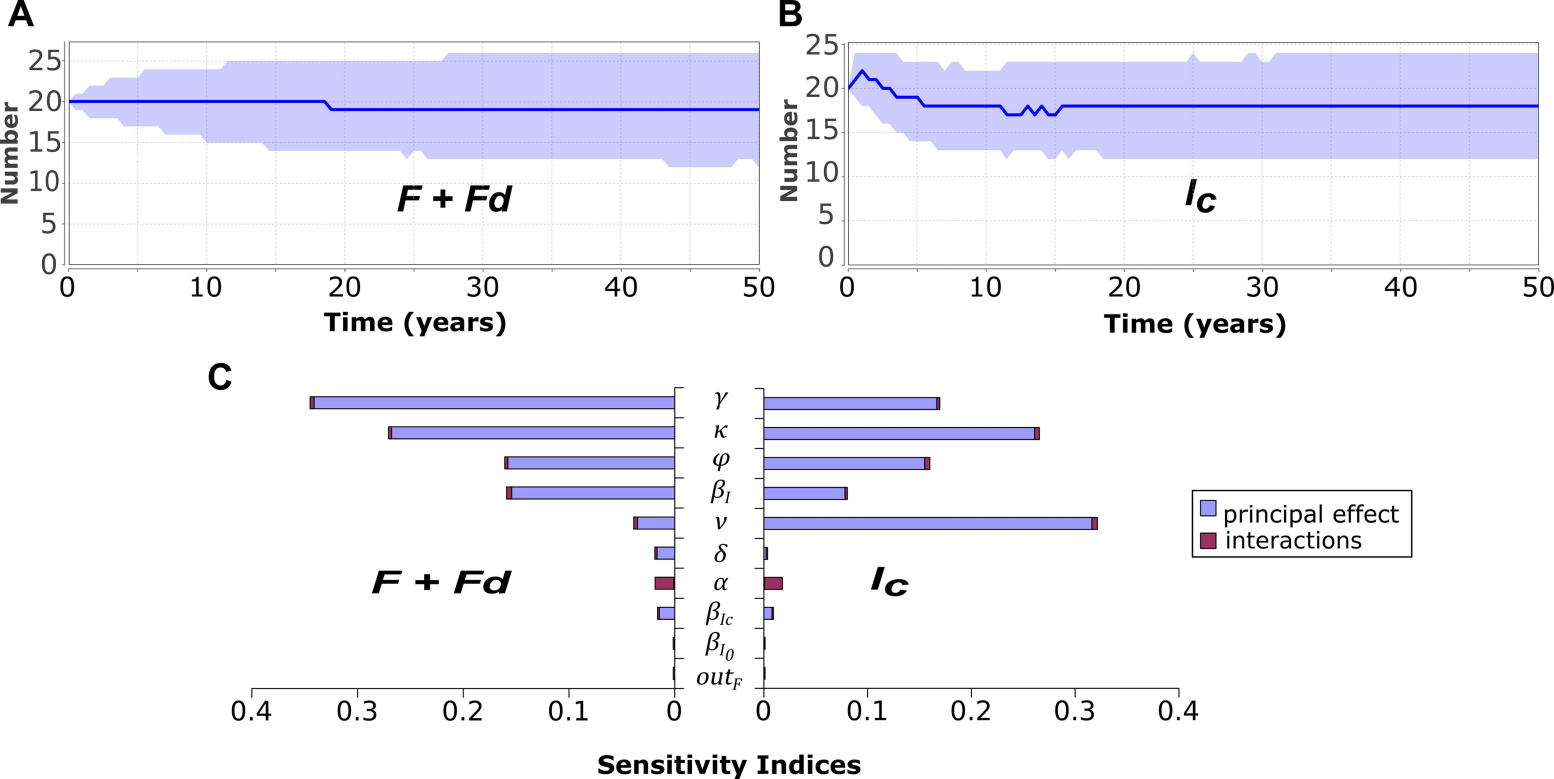
Behaviour of the epidemiological model of PRRS virus spread and control in a population of 50 herds when assuming no intervention from the social planner (5 000 replications). (A) Median number of virus-free herds (*F + Fd*). (B) Median number of infected controlled herds (*I*_*C*_). The 90% confidence interval [5^th^ percentile; 95^th^ percentile] was provided for each graph in light blue. (C) Contribution of each tested parameter to output variance (*β*_*I*_, $\beta_{I_{0}},\beta_{I_{C}}$, *out*,*γ*,*α*,*ν*,*κ*,*φ*,*δ*) after 50 years with the principal effect in blue and the effect associated to first order interactions in red (see Table 2 for parameter definition).

A description of the evolution of (*F + Fd*) and *I*_*C*_ numbers is provided in Fig 2A and 2C respectively. The median numbers of *I*_*C*_ and of (*F + Fd*) after 50 years were impacted (Fig 2C) by the probability of transition from *I*_*C*_ to *Fd* corresponding to the use of the individual measure of depopulation (*κ*), the protection due to biosecurity when in *Fd* (*γ*), the probability of transition from *I*_*C*_ to *I* corresponding to virus reintroduction due to lower biosecurity and vaccination (*φ*), and the transmission rate due to *I* herds (*β*_*I*_). The parameter *γ* impacted more (*F + Fd*) than *I*_*C*_ as it was directly associated to the risk of infection (leaving status *Fd*) and indirectly to the control (for *I*_*C*_). The probability of transition from *I* to *I*_*0*_ corresponding to the biosecurity and vaccination (*ν*) impacted mainly the mean number of *I*_*C*_ (Fig 2C) (which was expected as it concerns the control and not the virus clearance). Interactions barely influenced model output variations.

All of the parameters which can be impacted by actions of the social planner, except the transition from *F* to *Fd* (*α*), had a direct influence on both virus clearance and control within the system. Hence, if the social planner chose an action different from *None*, the prevalence of infected herds and of controlled herds should vary. The parameters that most impacted model outputs were all influenced by the social planner except the transmission rate due to *I* herds. As the median value of the number of (*F + Fd*) herds was near equilibrium (Fig 2A), we concluded that we had a good approximation of the value.

### Optimal computed policy

**Fig 3:**
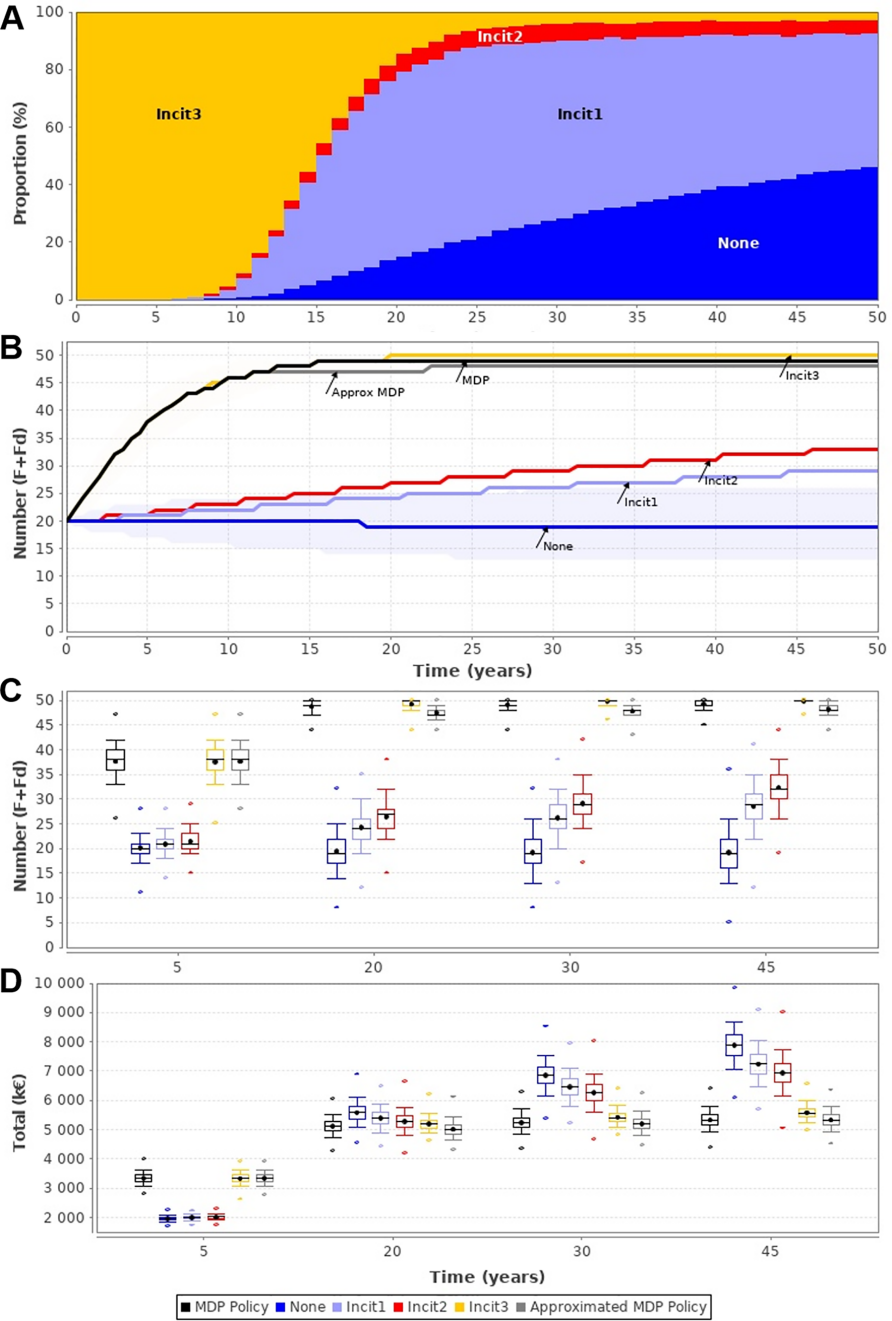
Model predictions when the social planner followed the optimal computed MDP policy, the approximated policy, or systematically used a single action. (A) Frequency of incentive actions over timewhile applying the optimal computed policy; (B) Variation of the median number of virus-free herds (*F + Fd*) when using the computed policy (MDP), the approximated one, or systematically a single action (*None*, *Incent1*, *Incent2*, *Incent3*); (C) Boxplot of the number of virus-free herds (*F + Fd*); (D) Boxplot of the discounted cumulated rewards summing losses and costs (in k€). (C-D): Dots corresponded to the minimal and maximal values, whisker limits are the 10 and 90 percentiles, box limits are the 25 and 75 percentiles, median and mean values are represented by a line and a cross, respectively. Boxplots are provided at 4 specific time points. Per scenario, 5,000 replications were performed.

Using π*, the social planner always used action *Incent3*during the first time-steps (Fig 3A). Then, according to the evolution of the system, the other actions also were used, action *Incent2*being barely used (Fig 3A). The use of π* reduced the disease prevalence (Fig 3B and 3C). After 45 years, the discounted cumulated rewards when using π* was lower than the one obtained if the social planner systematically used any other action (Fig 3D). After 50 years, using π* reducedthe rewards of 4% compared to the systematic use of action *Incent3*, and of 33% compared to the systematic use of action *None*. Such a result was expected as π* is defined as minimizing the discounted cumulative rewards. Nevertheless, the advantage of π* was observed after 18 years when comparing median values. Before 12 years, the discounted cumulative rewardswere similar to those obtained systematically using action *Incent3*because this action was used almost systematically in the first time-steps (Fig 3A). Although π* was more expensive than other actions over the first years (Fig 3D), we observed an advantage after 30 years as the prevalence and the discounted cumulative rewards were lower than those obtained with other actions (Fig 3C).

### Approximated policy

**Fig 4:**
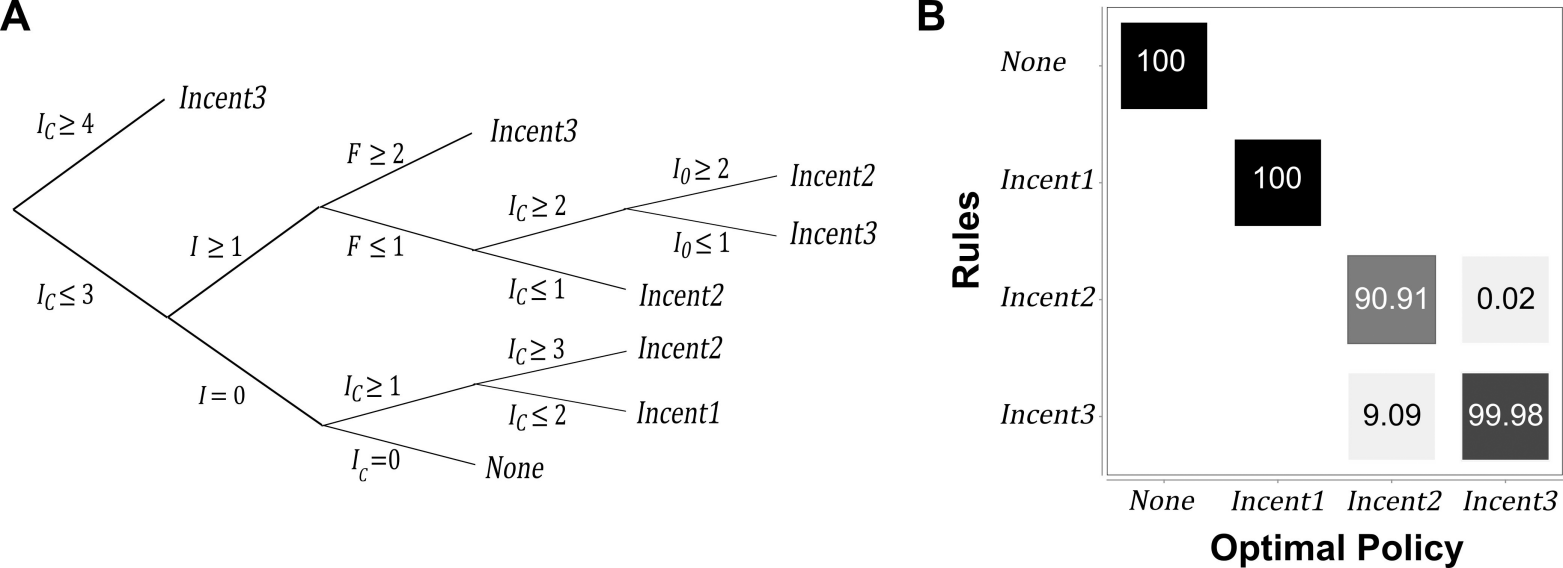
Approximated policy π*,^*approx*^ obtained using the C4.5 algorithm on the optimal policy π* when considering only available states. (A) Representation as a decision tree; (B) Concordance between π*,^*approx*^ and π* with π* as a reference (sum to 100 over a column).

The computed optimal policy π* was very complex. Working only on available states,we obtained 8 simple rules (Fig 4A). For a number of *I*_*C*_ herds higher than or equal to 4 (most states), *Incent3*was used (Fig 4A, top leaf). Otherwise, for a number of *I* herds equals 0, actions *None*, *Incent1*,and*Incent2*were used with respect to the number of *I*_*C*_ herds (Fig 4A, bottom leaf). If there was at least one *I* herd, actions *Incent2*and*Incent3*were used with respect to the number of *S*, *I*_*C*_, and *I*_*0*_herds (Fig 4A, bottom leaf). It has to be noted that on the branch with *I* ≥ 1 and *I*_*C*_ ≥ 2 herds, it is not known how many infected herds there are. Hence, it is not possible to conclude on the actual level of infection in the system. Actions predicted by π*,^*approx*^ were mostly the same as in π* (Fig 4B), and slightly differed only for states with actions *Incent2* and *Incent3* corresponding to less than 0.04% of misclassified actions among all of the available states. In addition, the epidemiological dynamics obtained following π*,^*approx*^ was close to the one obtained following π* both in terms of number of virus-free herds over time (Fig 3B and 3C) and in terms of cumulated cost (Fig 3D). Hence, the decision tree π*,^*approx*^ was a good approximation of the optimal policy π*, but providing a much more practical tool for decision-makers.

### Impact of uncertainty in parameter values on the model behavior

**Fig 5:**
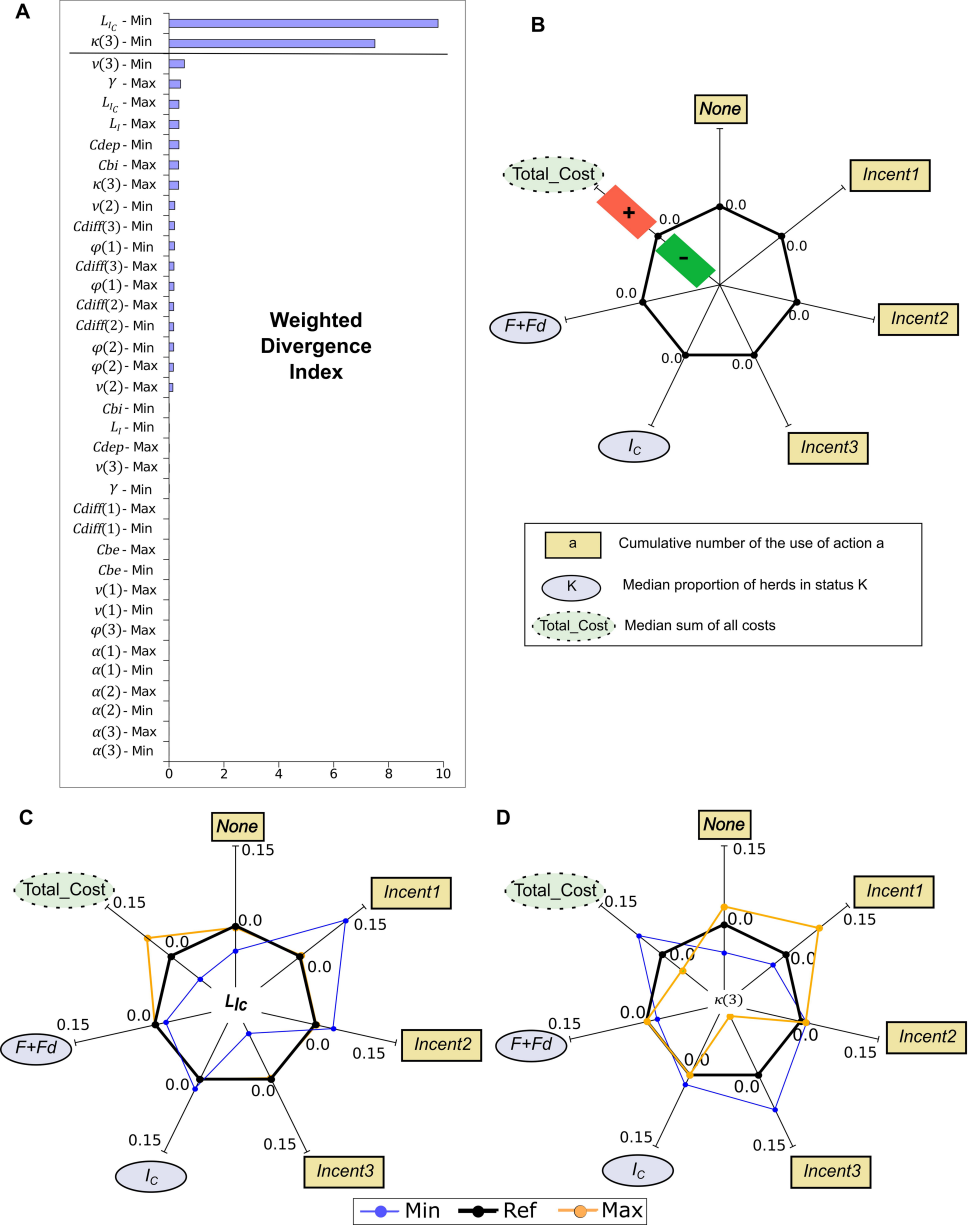
One-at-a-time sensitivity analysis of the optimal policy (Min and Max correspond to the minimal and maximal parameter values; Tables 2–4). (A) Weighted divergence index, parameters having different values according to the social planner’s action as shown by the number in parentheses (1 for *Incent1*, 2 for *Incent2*, and 3 for *Incent3*). (B) Reference spider plot provided for label definitions (no parameter variation): branches denote the considered outputs, curve position on a given branch describes the impact of varying the value of one parameter on this output. For each output, relative values are provided with respect to the reference scenario (thick line at 0.0). A value below (above) the thick line denotes a decrease (an increase) in the output (green '-' and red '+' areas, respectively). (C) Spider plot obtained when varying the cost of controlled infected herds (*L*_*Ic*_, Table 3) (D) Spider plot obtained when varying *k*(3), i.e. the transition probability from *I*_*C*_to *Fd*when action *Incent3* (depopulation) is retained by the social planner (Table 4).

Only two parameter variations induced a divergence between computed policies (Fig 5A): a decrease in the loss due to infection in *I*_*C*_ herds (*L*_*Ic*_), and a decrease in the probability of transition from *I*_*C*_ to *Fd* when action *Incent3*was considered (noted *κ*(3)). A decreased value of *L*_*Ic*_ induced an increase in the cumulative use of action *Incent1*, a decrease in the cumulative use of action *Incent3*, and a decrease in the clearance level corresponding to an increase of the infection prevalence (Fig 5C). *L*_*Ic*_as a cost impacted the median total cost when both decreasing and increasing (Fig 5C). For the variation of the transition probability between *I*_*C*_ and *Fd* (*κ*(3)), the use of each action and the total costs were impacted (Fig 5D), its increase particularly decreasing the use of *Incent3*while increasing the use of *None* and *Incent1*.

Variations of other parameter valuesbarely impacted π*, resulting in only few variations of simulated results. Obviously, if a cost varied, the total cost was modified as expected. Parameters inducing no variation of π* on available states were the cost of external biosecurity (*Cbe*), the transition probability between *F* and *Fd* (*α*irrespective of the action of the social planner, the transition probability between *I* and *I*_*0*_ (*ν*) for action *Incent1*only, the transition probability between *I*_*C*_ and *I* (*φ*) for action *Incent3*only, and the fixed cost (*Cdiff*) for action *Incent1*.

## Discussion

In this paper, we proposed a framework which can be used by a decision-maker acting as a social planner to identify an adaptive strategy consisting of incentives in order to optimise the net financial benefit at the collective scale for a group of farmers. We applied this framework for the control of PRRS, an endemic non-notifiable disease. The computed policy providing better net financial benefit was adaptive. It illustrated the potential of adaptive approach to propose an optimal dynamic strategy. Even if some incentives were expensive, the optimisation over a long horizon took into account the incentive benefits to balance these high costs. This framework is a tool to help a social planner to define collective schemes, provided that the social planner is accurately informed about the health status of the herds.As an example, for PRRS, the Morrison’s Swine Health Monitoring Project is conducted on a convenience sample of 910 herds for which diagnostic status is reported weekly [41]. To be used in field conditions (and thus as a tool for decision support), the framework should be specified according to the aim of the collective decision-maker by defining the corresponding objective function, the horizon for optimisation, the incentive levels, and the impact of farmers’ response to incentives. As the framework can become verycomplex for use by a decision maker (“curse of dimensionality”), a compromise should be found between realism and simplification. In such a case, the decision-maker should be involved in the modelling phase to inform the model assumptions and interpret results. One perspective to this study is to implement a participative research project in which stakeholders would be involved in order to incentivize them to test and improve our framework.

The definition of the objective function is one of the most important components. According to the objective function, the MDP policies can be different as shown in [20]. In our case study, we optimised the net financial benefit at the collective scale. The disease prevalence which is a result of interest for epidemiologists was introduced indirectly in the objective function as each infected farm induced losses and control costs for the decision-maker. Even if the optimisation was based on losses and control costs, the computed policy in our case study had a positive impact on disease prevalence. On the other hand, decision-makers may be interested in optimising simultaneously several objectives (for instance decreasing both costs and disease prevalence). For optimising more than one criterion at a time, the framework has to be adapted.The method used here to compute the optimal policy does not allowthe use of combined criterion. However, relevant algorithms to compute the optimal policy in such a case can be found in the literature [42, 43].

It is not straightforward to anticipate when a given disease situation will show a large impact of an adaptive policy compared to a fixed one.Of course, it is expected to vary with the pathosystem, as well as with the considered control options and incentives. As regards our case study on PRRS virus, the difference between the adaptive policy and a fixed one is small as regards saved euros (4%) but large as regards acceptability of the collective scheme. Indeed, eradication can almost be achieved without the need of implementing forever a very constraining action (depopulation). This action is already known by health managers as the main one that can impact PRRS herd prevalence.It is also known by that eradication of such an endemic disease cannot be achieved in a few years only. However, when to stop a control action such as depopulation was unknown. We highlighted that 10 years after the start of depopulation implementation in detected infected herds, this action could be progressively stopped and replaced by lighter ones for the next 10 years without impairing the large reduction in prevalence almost achieving eradication which would have been obtained with a fixed action on the same duration.

Another main component of the framework to be decided with the decision-maker is the horizon of optimisation. In our case study, we used an infinite time horizon. It was retained as PRRS virus is highly prevalent [28] and often no eradication was looked for at first, leading us to assume that the disease may persist in the long run. The optimal policy computed with the infinite time horizon can be acceptable even for a finite horizon if the finite horizon is long enough and if an accurate discount factor is used. In our case study, the total cost obtained with the optimal policy was better than others after 25 years onward (Fig 3D). Nevertheless, the social planner may choose to reach an objective before a specified time (finite horizon). In our framework, the computation of the optimal policy should be modified to optimise only over a finite horizon. Meanwhile, when computing a policy over a finite horizon, it has to be assumed that the system does not exist after the end of the optimisation horizon, which may induce some drawbacks when the policy is used for long-term system [27]. For example, some incentives may avoid the steps just before the end of the horizon if their impact can be observed only a few time-steps after. To avoid such drawbacks, a rolling horizon approach has been proposed [44] but optimality is not guaranteed [45]. The decision-maker thus should define the horizon having knowledge about advantages and drawbacks. Regarding our framework, we used the Value Iteration algorithm [26] which can be used for computing both finite and infinite horizons and adapted for computing rolling horizon.

To render the policy usable in field conditions, we proposed a way to approximate the optimal computed policy which was far too complex for practical purposes. For a decision-maker, having simple decision rules is important for practical use in field conditions as pointed out by Pichancourt*et al*. [46]. It was possible to transform the policy into decision rules (*if … then … else* …), using for example the approach of Gil *et al*. [47]. However, due to the complexity of the optimal computed policy in our case study, the number of obtained rules using such an approach would have been far too high to be usable under field conditions. Therefore, we used a method developed in the area of data mining for the analysis of large volume of data [39] and produced a decision tree having only eight rules. We showed that this approximation was relevant regarding retained actions. In [48], an approximated policy was also computed to simplify their complex policy. As in our paper, the agreement was verified only in terms of actions, not on the value of the objective function. The optimality thus is not guaranteed. Steimle and Denton [49] considered that proposing a simple approximated policy although sub-optimal is a way to enhance the application of the policy by decision-makers.

The sensitivity analysis is a crucial step in modelling work to assess the impact of parameter uncertainty on model outputs [37]. Performing a sensitivity analyses helps the decision-maker to identify knowledge gaps impacting predictions. Here, two analyses have been performed. The first one was based on the model without incentive (always doing nothing), which allows the decision-maker to identify possible policy instruments. The second one highlighted parameters impacting the predicted optimal policy, illustrating for the decision-maker the impact of parameter uncertainty on the policy. More precise values are needed for parameters corresponding to pathogen spread or to an economic value (for example in our case study, parameter *L*_*Ic*_ corresponding to the losses for *Ic* herds). In addition, some identified parameters correspond to the impact of the farmer’s response to a decision-maker action (incentive), such as the transition from *I*_*C*_ to *Fd*which corresponds to depopulation when the social planner retains action *Incent3*.For such parameters, a knowledge on how farmers would respond to incentives is needed. To estimate farmers' responses, either data on previous use of incentives can be explored [50] or behaviour experiments can be proposed such as in [51]. Moreover, global sensitivity analysis—in which the values of all parameters simultaneously vary—can be done only for the first analysis. For the second one, the approach proposed by [52] evaluated the result confidence both on policy and objective function. In this approach, parameters would be considered either one by one or jointly. In our case study, we did only scenario analysis corresponding to a one-at–a-time (OAT) analysis with two values for each parameter due to the high computing time per parameter set.

Our framework is a novel contribution in terms of a decision-making support that can be applied to animal disease control situations. In the special case of non-notifiable diseases, control decisions are made by farmers based on the risk of their herd being infected and on disease consequences, sometimes in interaction with a social planner. In animal health literature, models focused on individual decision and scenarios comparing collective incentive schemes [12, 53]. These models did not consider an optimal decision of the social planner,but only the impact of incentives on farmer’s decisions. Instead, we proposed to consider interactions between farmers’ decisions and collective actions through incentives. Our framework provides a practical tool for decision makers to evaluate *a priori*their policy under a variety of epidemiological situations and incentive levels.

## Supporting information

S1 File Code. This archive contains the data and the Java code required to run the MDP model.
(ZIP)

S2 File Results. This archive contains the data used in the results section of the paper.
(ZIP)

## References

[1] Ekboir JM. The role of the public sector in the development and implementation of animal health policies. Prev Vet Med. 1999;40(2):101–15. 10384947 10.1016/s0167-5877(99)00015-x

[2] Beaunée G, Vergu E, Ezanno P. Modelling of paratuberculosis spread between dairy cattle farms at a regional scale. Vet Res. 2015;46(1):111. 26407894 10.1186/s13567-015-0247-3 PMC4583165

[3] Qi L, Vergu E, Dutta BL, Joly A, Ezanno P. Proximity contacts and trade movements drive the spread of endemic pathogens in heterogeneous managed metapopulation. Epidemics (submitted).

[4] Pandit P, Hoch T, Ezanno P, BeaudeauFo, Vergu E. Spread of Coxiella burnetii between dairy cattle herds in an enzootic region: modelling contributions of airborne transmission and trade. Vet Res. 2016;47(1):48. 27048416 10.1186/s13567-016-0330-4 PMC4822316

[5] Nusinovici S, Frössling J, Widgren S, Beaudeau F, Lindberg A. Q fever infection in dairy cattle herds: increased risk with high wind speed and low precipitation. Epidemiol Infect. 2015;143(15):3316–26. doi: 10.1017/S0950268814003926 25783480 10.1017/S0950268814003926 PMC4594051

[6] Charron MVP, Kluiters G, Langlais M, Seegers H, Baylis M, Ezanno P. Seasonal and spatial heterogeneities in host and vector abundances impact the spatiotemporal spread of bluetongue. Vet Res. 2013;44(1):44. 23782421 10.1186/1297-9716-44-44 PMC3701505

[7] Woodroffe R, Donnelly CA, Jenkins HE, Johnston WT, Cox DR, Bourne FJ, et al Culling and cattle controls influence tuberculosis risk for badgers. Proc Natl Acad Sci USA. 2006;103(40):14713–7. doi: 10.1073/pnas.0606251103 17015843 10.1073/pnas.0606251103 PMC1586183

[8] Rossi G, Smith RL, Pongolini S, Bolzoni L. Modelling farm-to-farm disease transmission through personnel movements: from visits to contacts, and back. Sci Rep. 2017;7. 10.1038/s41598-017-02567-6 PMC5443770 28539663

[9] Klein E, Laxminarayan R, Smith DL, Gilligan CA. Economic incentives and mathematical models of disease. Environment and development economics. 2007;12(05):707–32.

[10] Ibuka Y, Li M, Vietri J, Chapman GB, Galvani AP. Free-riding behavior in vaccination decisions: An experimental study. PloSOne. 2014;9(1):e87164. 10.1371/journal.pone.0087164 PMC3901764 24475246

[11] Bauch CT, Galvani AP, Earn DJD. Group interest versus self-interest in smallpox vaccination policy. Proc Natl Acad Sci USA. 2003;100(18):10564–7. doi: 10.1073/pnas.1731324100 12920181 10.1073/pnas.1731324100 PMC193525

[12] Rat-Aspert O, Fourichon C. Modelling collective effectiveness of voluntary vaccination with and without incentives. Prev Vet Med. 2010;93(4):265–75. doi: 10.1016/j.prevetmed.2009.11.004 20022648 10.1016/j.prevetmed.2009.11.004

[13] Manfredi P, Della Posta P, d’Onofrio A, Salinelli E, Centrone F, Meo C, et al Optimal vaccination choice, vaccination games, and rational exemption: an appraisal. Vaccine. 2009;28(1):98–109. doi: 10.1016/j.vaccine.2009.09.109 19836477 10.1016/j.vaccine.2009.09.109

[14] Kristensen AR. A general software system for Markov decision processes in herd management applications. Comput Electron Agric. 2003;38(3):199–215.

[15] Kristensen AR. From biological models to economic optimization. Prev Vet Med. 2015;118(2):226–37. 25496776 10.1016/j.prevetmed.2014.11.019

[16] Chadès I, Martin TG, Nicol S, Burgman MA, Possingham HP, Buckley YM. General rules for managing and surveying networks of pests, diseases, and endangered species. Proc Natl Acad Sci USA. 2011;108(20):8323–8. doi: 10.1073/pnas.1016846108 21536884 10.1073/pnas.1016846108 PMC3100963

[17] Johnson FA, Breininger DR, Duncan BW, Nichols JD, Runge MC, Williams BK. A Markov decision process for managing habitat for Florida scrub-jays. J Fish WildlManag. 2011;2(2):234–46.

[18] Merl D, Johnson LR, Gramacy RB, Mangel M. A statistical framework for the adaptive management of epidemiological interventions. PloS One. 2009;4(6):e5807 doi: 10.1371/journal.pone.0005807 19503812 10.1371/journal.pone.0005807 PMC2688756

[19] Yaesoubi R, Cohen T. Generalized Markov models of infectious disease spread: A novel framework for developing dynamic health policies. Eur J Oper Res. 2011;215(3):679–87. doi: 10.1016/j.ejor.2011.07.016 21966083 10.1016/j.ejor.2011.07.016 PMC3182455

[20] Mason JE, Denton BT. A comparison of decision-maker perspectives for optimal cholesterol treatment. IBM J Res Dev. 2012;56(5):8: 1–8: 12.

[21] Ge L, Mourits MCM, Kristensen AR, Huirne RBM. A modelling approach to support dynamic decision-making in the control of FMD epidemics. Prev Vet Med. 2010;95(3):167–74. 20471708 10.1016/j.prevetmed.2010.04.003

[22] Viet AF, Jeanpierre L, Bouzid M, Mouaddib AI. Using Markov Decision Processes to define an adaptive strategy to control the spread of an animal disease. Comput Electron Agric. 2012;80:71–9.

[23] Nieuwenhuis N, Duinhof TF, Van Nes A. Economic analysis of outbreaks of porcine reproductive and respiratory syndrome virus in nine sow herds. Vet Rec. 2012;170(9):225 doi: 10.1136/vr.100101 22238201 10.1136/vr.100101

[24] Holtkamp DJ, Kliebenstein JB, Neumann EJ, Zimmerman JJ, Rotto HF, Yoder TK, et al Assessment of the economic impact of porcine reproductive and respiratory syndrome virus on United States pork producers. J Swine Health Prod. 2013;21(2):72–84.

[25] Gersovitz M, Hammer JS. The economical control of infectious diseases. Econ. J. 2004;114(492):1–27.

[26] Puterman ML. Markov decision processes: discrete stochastic dynamic programming: John Wiley & Sons; 2005.

[27] Marescot L, Chapron G, Chadès I, Fackler PL, Duchamp C, Marboutin E, et al Complex decisions made simple: a primer on stochastic dynamic programming. Methods EcolEvol. 2013;4(9):872–84.

[28] Pileri E, Mateu E. Review on the transmission porcine reproductive and respiratory syndrome virus between pigs and farms and impact on vaccination. Vet Res. 2016;47(1):108 doi: 10.1186/s13567-016-0391-4 27793195 10.1186/s13567-016-0391-4 PMC5086057

[29] Holtkamp DJ, Polson DD, Torremorell M, Classen DM, Becton L, Henry S, et al Terminology for classifying swine herds by porcine reproductive and respiratory syndrome virus status. J Swine Health Prod. 2011;19(1):44–56. 22138772

[30] Corzo CA, Mondaca E, Wayne S, Torremorell M, Dee S, Davies P, et al Control and elimination of porcine reproductive and respiratory syndrome virus. Virus Res. 2010;154(1):185–92. 20837071 10.1016/j.virusres.2010.08.016

[31] Valdes-Donoso P, Jarvis LS, Wright D, Alvarez J, Perez AM. Measuring progress on the control of porcine reproductive and respiratory syndrome (PRRS) at a regional level: the minnesota N212 regional control project (Rcp) as a working example. PloSOne. 2016;11(2):e0149498. 10.1371/journal.pone.0149498 PMC4760934 26895148

[32] Le Potier MF, Blanquefort P, Morvan E, Albina E. Results of a control programme for the porcine reproductive and respiratory syndrome in the French "˜Pays de la Loire" region. Vet Microbiol. 1997;55(1):355–60. 9220633 10.1016/s0378-1135(96)01318-1

[33] Perez AM, Davies PR, Goodell CK, Holtkamp DJ, Mondaca-Fernandez E, Poljak Z, et al Lessons learned and knowledge gaps about the epidemiology and control of porcine reproductive and respiratory syndrome virus in North America. J Am Vet Med Assoc. 2015;246(12):1304–17. doi: 10.2460/javma.246.12.1304 26043128 10.2460/javma.246.12.1304

[34] Begon M, Bennett M, Bowers RG, French NP, Hazel SM, Turner J. A clarification of transmission terms in host-microparasite models: numbers, densities and areas. Epidemiol Infect. 2002;129(01):147–53. 12211582 10.1017/s0950268802007148 PMC2869860

[35] Hoch T, Fourichon C, Viet AF, Seegers H. Influence of the transmission function on a simulated pathogen spread within a population. Epidemiol Infect. 2008:1374–82. doi: 10.1017/S095026880700979X 18062825 10.1017/S095026880700979X PMC2870731

[36] Flanagan D. Java in a Nutshell: " O'Reilly Media, Inc." 2005.

[37] Saltelli A, Chan K, Scott EM. Sensitivity analysis: Wiley New York; 2000.

[38] R Development Core Team. R: A language and environment for statistical computing R Foundation for Statistical Computing Vienna, Austria ISBN 3-900051-07-0, URL http://www.R-project.org. 2010.

[39] Wu X, Kumar V, Quinlan JR, Ghosh J, Yang Q, Motoda H, et al Top 10 algorithms in data mining. Knowl Inf Syst. 2008;14(1):1–37.

[40] Carrizosa E, Morales DR. Supervised classification and mathematical optimization. ComputOper Res. 2013;40(1):150–65.

[41] Silva GS, Schwartz M, Morrisson RB, Linhares DCL. Monitoring breeding herd production data to detect PRRSV outbreaks. Prev Vet Med. 2017;148:89–93. doi: 10.1016/j.prevetmed.2017.10.012 29157378 10.1016/j.prevetmed.2017.10.012

[42] Boussard M, Bouzid M, Mouaddib A-I, editors. Multi-criteria decision making for local coordination in multi-agent systems. 19th IEEE International Conference on Tools with Artificial Intelligence, 2007.

[43] Chatterjee K, Majumdar R, Henzinger TA, editors. Markov decision processes with multiple objectives. Annual Symposium on Theoretical Aspects of Computer Science; 2006: Springer.

[44] Hernandez-Lerma O, Lasserre JB. Value iteration and rolling plans for Markov control processes with unbounded rewards. J Math Anal Appl. 1993;177(1):38–55.

[45] Alden JM, Smith RL. Rolling horizon procedures in nonhomogeneous Markov decision processes. Oper Res. 1992;40(3-supplement-2):S183–S94.

[46] Pichancourt JB, Chadès I, Firn J, van Klinken RD, Martin TG. Simple rules to contain an invasive species with a complex life cycle and high dispersal capacity. J Appl Ecol. 2012;49(1):52–62.

[47] Gil AJ Hermann M, Salzer G, Zanuttini B. Efficient algorithms for description problems over finite totally ordered domains. SIAM J Comput. 2008;38(3):922–45.

[48] Nicol S, Sabbadin R, Peyrard N, Chadès I. Finding the best management policy to eradicate invasive species from spatial ecological networks with simultaneous actions. J Appl Ecol. 2017:Forthcoming.

[49] Steimle LN, Denton BT. Markov decision processes for screening and treatment of chronic diseases Markov Decision Processes in Practice: Springer; 2017 p. 189–222.

[50] Nöremark M, Lindberg A, Vagsholm I, Lewerin SS. Disease awareness, information retrieval and change in biosecurity routines among pig farmers in association with the first PRRS outbreak in Sweden. Prev Vet Med. 2009;90(1):1–9. 19376601 10.1016/j.prevetmed.2009.03.008

[51] Chapman GB, Li M, Vietri J, Ibuka Y, Thomas D, Yoon H, et al Using game theory to examine incentives in influenza vaccination behavior. Psychol Sci. 2012;23(9):1008–15. doi: 10.1177/0956797612437606 22810166 10.1177/0956797612437606

[52] Chen Q, Ayer T, Chhatwal J. Sensitivity Analysis in Sequential Decision Models: A Probabilistic Approach. Med Decis Making. 2017;37(2):243–52. doi: 10.1177/0272989X16670605 27681992 10.1177/0272989X16670605

[53] Rat-Aspert O, Krebs S, editors. Individual and collective management of endemic animal diseases: an economic approach. 2012 Conference, August 18–24, 2012, Foz do Iguacu, Brazil; 2012: International Association of Agricultural Economists.

